# Deep Learning Predicts Heart Failure With Preserved, Mid-Range, and Reduced Left Ventricular Ejection Fraction From Patient Clinical Profiles

**DOI:** 10.3389/fcvm.2021.755968

**Published:** 2021-11-22

**Authors:** Mohanad Alkhodari, Herbert F. Jelinek, Angelos Karlas, Stergios Soulaidopoulos, Petros Arsenos, Ioannis Doundoulakis, Konstantinos A. Gatzoulis, Konstantinos Tsioufis, Leontios J. Hadjileontiadis, Ahsan H. Khandoker

**Affiliations:** ^1^Department of Biomedical Engineering, Healthcare Engineering Innovation Center (HEIC), Khalifa University, Abu Dhabi, United Arab Emirates; ^2^Department of Biomedical Engineering, Biotechnology Center (BTC), Khalifa University, Abu Dhabi, United Arab Emirates; ^3^Chair of Biological Imaging, Center for Translational Cancer Research (TranslaTUM), Technical University of Munich, Munich, Germany; ^4^Institute of Biological and Medical Imaging, Helmholtz Zentrum München, Neuherberg, Germany; ^5^Department for Vascular and Endovascular Surgery, Rechts der Isar University Hospital, Technical University of Munich, Munich, Germany; ^6^DZHK (German Centre for Cardiovascular Research), Partner Site Munich Heart Alliance, Munich, Germany; ^7^First Cardiology Department, School of Medicine, “Hippokration” General Hospital, National and Kapodistrian University of Athens, Athens, Greece; ^8^Department of Electrical Engineering and Computer Science, Khalifa University, Abu Dhabi, United Arab Emirates; ^9^Department of Electrical and Computer Engineering, Aristotle University of Thessaloniki, Thessaloniki, Greece

**Keywords:** heart failure, coronary artery disease, left ventricular ejection fraction, clinical profiles, demographical and clinical information, radial visualization, machine and deep learning

## Abstract

**Background:** Left ventricular ejection fraction (LVEF) is the gold standard for evaluating heart failure (HF) in coronary artery disease (CAD) patients. It is an essential metric in categorizing HF patients as preserved (HFpEF), mid-range (HFmEF), and reduced (HFrEF) ejection fraction but differs, depending on whether the ASE/EACVI or ESC guidelines are used to classify HF.

**Objectives:** We sought to investigate the effectiveness of using deep learning as an automated tool to predict LVEF from patient clinical profiles using regression and classification trained models. We further investigate the effect of utilizing other LVEF-based thresholds to examine the discrimination ability of deep learning between HF categories grouped with narrower ranges.

**Methods:** Data from 303 CAD patients were obtained from American and Greek patient databases and categorized based on the American Society of Echocardiography and the European Association of Cardiovascular Imaging (ASE/EACVI) guidelines into HFpEF (EF > 55%), HFmEF (50% ≤ EF ≤ 55%), and HFrEF (EF < 50%). Clinical profiles included 13 demographical and clinical markers grouped as cardiovascular risk factors, medication, and history. The most significant and important markers were determined using linear regression fitting and Chi-squared test combined with a novel dimensionality reduction algorithm based on arc radial visualization (ArcViz). Two deep learning-based models were then developed and trained using convolutional neural networks (CNN) to estimate LVEF levels from the clinical information and for classification into one of three LVEF-based HF categories.

**Results:** A total of seven clinical markers were found important for discriminating between the three HF categories. Using statistical analysis, diabetes, diuretics medication, and prior myocardial infarction were found statistically significant (*p* < 0.001). Furthermore, age, body mass index (BMI), anti-arrhythmics medication, and previous ventricular tachycardia were found important after projections on the ArcViz convex hull with an average nearest centroid (NC) accuracy of 94%. The regression model estimated LVEF levels successfully with an overall accuracy of 90%, average root mean square error (RMSE) of 4.13, and correlation coefficient of 0.85. A significant improvement was then obtained with the classification model, which predicted HF categories with an accuracy ≥93%, sensitivity ≥89%, 1-specificity <5%, and average area under the receiver operating characteristics curve (AUROC) of 0.98.

**Conclusions:** Our study suggests the potential of implementing deep learning-based models clinically to ensure faster, yet accurate, automatic prediction of HF based on the ASE/EACVI LVEF guidelines with only clinical profiles and corresponding information as input to the models. Invasive, expensive, and time-consuming clinical testing could thus be avoided, enabling reduced stress in patients and simpler triage for further intervention.

## Introduction

Heart failure (HF) is a chronic and progressive pathologic state characterized by the inability of the heart to pump an adequate amount of blood to supply tissues with nutrients via the systemic circulation (1). Several conditions, such as coronary artery disease (CAD) and arterial hypertension, are considered major causes of HF progression (2, 3). According to the European Society of Cardiology (ESC), more than 26 million people around the world suffer from HF caused by CAD (4). Furthermore, the World Health Organization (WHO) estimates that HF accounts for more than 7.2 million deaths annually worldwide (3).

The systolic function of the heart, as indicated by the left ventricular ejection fraction (LVEF), is significantly decreased in HF. LVEF refers to the amount (%) of oxygenated blood pumped out of the left ventricle at each contraction of the heart (5, 6). It is considered an important diagnostic metric in evaluating the progression of HF, especially at early stages. Based on the LVEF, HF can be classified according to the American Society of Echocardiography and the European Association of Cardiovascular Imaging (ASE/EACVI) (7–9) into three main categories: heart failure with preserved ejection fraction (HFpEF) with an EF above 55%, heart failure with mid-range ejection fraction (HFmEF) with an EF between 50 and 55%, and heart failure with reduced ejection fraction (HFrEF) with an EF below 50%. The narrower range for the HFmEF category is considered as a variable criteria for this group in accordance to the etiology of HF. Other guidelines including the ESC (10) recommend different cut-off values for classification of HF, with a cut-off for HFrEF as low as 40%. The literature suggests that there are no strict rules and that the treatment is loosely associated with LVEF and clinical presentation. However, patients in the mid-range group between 40 and 49% based on the ESC guidelines show that 90% of patients either improved or deteriorated, whilst only 10% of cases remained unchanged (11).

Accurate LVEF-based assessment of HF therefore poses substantial challenges to clinicians (8, 9, 12). HFpEF, despite covering half of all patients with HF, is not yet well-understood and remains frequently undetected due to similarities in symptoms and adverse outcomes with HFrEF and, to a lesser extent, HFmEF (12, 13). Furthermore, HFmEF represents one-fifth of the HF population and remains ambiguous, as its pathogenesis was observed to be more similar to that of HFrEF and rather different from HFpEF depending on the guidelines applied. This raises the question of whether it should be considered a transient entity between HFpEF and HFrEF or a distinct entity on its own (14–16). Therefore, additional research is needed to investigate the effectiveness of LVEF-based categorization of HF patients. According to recently published studies, clinical profiles of patients allow for the discrimination between the three HF categories, especially the presence of comorbidities and quality of life based on the ESC guidelines (16–21). Based on these clinical results, HFmEF patients were found to fall between HFpEF and HFrEF while more closely resembling HFpEF (22, 23). Additionally, they were more likely to be younger than HFpEF and more prone to diabetes and hypertension than HFrEF (20, 24). Thus, further studies on a larger cohort of patients are still required to understand how demographical and clinical characteristics are associated with each HF category defined by clinically measured ejection fraction, especially in terms of optimizing treatment options to improve stratification and risk management of patients.

Most recently, machine learning has been widely implemented in medical research to assist in HF assessment through clinical information (25–30). In addition, several studies have employed machine learning, including unsupervised clustering, to identify and characterize sub-groups of HFpEF from patient clinical profiles (31–34). However, there is still a limited knowledge on the complex relationship between demographical and clinical information and the three LVEF-based HF categories. In addition, it would be highly appreciated to offer a promising alternative tool to echocardiography for LVEF assessment which does not require the highly specialized knowledge and expensive equipment. In this vein, machine learning, including deep learning, can be essential in understanding the complicated clinical characteristics included in patient records leading to a better HF assessment. Therefore, in this study, we sought to investigate the ability of deep learning-based trained models in estimating LVEF levels as well as predicting HF categories from patient demographic and clinical information only in line with the ASE/EACVI guidelines. No previous studies have employed deep learning for analyzing HF categories associated with clinical profiles and LVEF. Thus, we developed trained models that could be capable of automatically providing assistance in clinical decision making in HF assessment based on LVEF levels. To prevent training the models using arbitrary or biased clinical variables, we ensured the following two steps: first, we investigated the statistical significance of each variable in discriminating between the three categories, and second, we followed a novel dimensionality reduction technique based on radial visualization to observe the best variables in characterizing and separating each LVEF-based HF category. We report the performance of the developed models that were trained based on the most important clinical variables to discuss the importance of deep learning in HF analysis based on LVEF as well as to elaborate on the significance of these clinical variables within patient profiles in differentiating between the three HF categories.

## Materials and Methods

### Dataset and Patients Enrollment

Two datasets that contain clinical information of American and Greek patient cohorts were included in this study. Both datasets included patients with HF, more specifically CAD, with ages between 33 and 88 years (*n* = 303). These patients were divided into 129 HFpEF, 92 HFmEF, and 82 HFrEF according to the ASE/EACVI guidelines.

The American patient cohort was obtained from the archives of the Intercity Digital Electrocardiography (ECG) Alliance (IDEAL) study of the University of Rochester Medical Center Telemetric and Holter ECG Warehouse (THEW) (35). The database enrollment protocol was conducted according to Title 45, U.S. Code of Federal Regulations, Part 46, protection of human subjects (revised: November 13, 2001–effective: December 13, 2001) and in accordance with the Declaration of Helsinki. Furthermore, the research subject review board of the University of Rochester approved the IDEAL protocol (36). All patients provided a signed consent before participating in the study. The eligibility criteria to enroll in the IDEAL study included: (1) having either an evidence of previous MI or an exercise induced ischemia; (2) being in stable phase of ischemic heart disease at least 2 months after the last event; (3) not diagnosed with a congenital heart failure; and (4) being in sinus rhythm. Furthermore, all patients with dilated cardiomyopathy (left ventricular diameter (LVD) > 60 mm and EF <40%), congenital heart failure (CHF), coronary artery bypass grafting (CABG) surgery, non-sinus rhythm, and any cerebral, severe hepatic, or malignancy diseases were excluded from the study. A total of 199 patients were included from the IDEAL study. Out of these patients, HFpEF (*n* = 106), HFmEF (*n* = 46), and HFrEF (*n* = 47) categories were grouped based on the aforementioned ASE/EACVI guidelines.

The Greek patient cohort was obtained from the PRESERVE EF study with patients enrolled across seven cardiology departments in Greece (37). The protocol of the study was approved by the ethics committee at each cardiology department and was endorsed by the Hellenic Society of Cardiology. A database was created and is maintained by the Hellenic Society of Cardiology (38). All patients signed a consent form prior to enrollment in the study at each cardiology department. The eligibility criteria for patient enrollment included: (1) having a post-angiographically proven MI of at least 40 days after the event or 90 days after any CABG surgeries, if applicable; (2) being revascularized; (3) being not revascularized but without evidence of any active ischemia in previous the 6 months; and (4) following optimal and tolerated medical therapy. Furthermore, any patient with a secondary prevention indication for implantable cardioverter defibrillator (ICD) implantation, permanent pacemaker, persistent, long-standing persistent, and permanent atrial fibrillation, any neurological symptoms of syncope or pre-syncope within the last 6 months, and presence of any systemic illnesses such as liver failure, renal diseases, rheumatic diseases, thyroid dysfunction, and cancer was excluded from the study. Overall, a total of 104 patients were obtained from the PRESERVE EF study. These patients were distributed as 23 HFpEF, 46 HFmEF, and 35 HFrEF based on the ASE/EACVI guidelines.

### Demographic and Clinical Markers

Both datasets included demographic and clinical information. Provided information was initially grouped into cardiovascular risk factors, cardiovascular medication, and cardiovascular history. As cardiovascular risk factors, age (years), sex (male—female), body mass index (BMI, kg/m^2^), smoking (yes—no), diabetes (yes–no), and hypertension (yes—no) were the recorded markers. As cardiovascular medication, beta-blockers (yes—no), angiotensin-converting enzyme inhibitors (ACE-inhibitors, yes—no), anti-arrhythmics (yes—no), and diuretics (yes—no) were selected. Lastly, cardiovascular history included the presence of any previous angina pectoris (AP, yes—no), ventricular tachycardia (VT, yes—no), and myocardial infractions (Prior MI, yes—no).

### Statistical Data Analysis

The statistical analysis was carried out using Student's *t*-test based on linear regression fitting (39), where the significance of each variable was evaluated based on the corresponding *p*-value measurement, with a *p*-value below 0.05 indicating significance. A chi-squared (χ^2^) test (40) was applied to examine which clinical variables were most important and highly dependent on individual LVEF categories. In this test, an important feature with a high score indicates a significant difference (*p* < 0.05) in discriminating between the three LVEF categories.

### Multivariate Data Visualization

Patient information, being high-dimensional data, requires further projections into a low-dimensional space (dimensionality reduction) for multivariate visual inspection, and for clustering and pattern recognition purposes. A modified version of the original radial visualization (RadViz) algorithm (41, 42) proposed by Van Long (43), based on arc representation of variables (ArcViz) rather than point or line representations, was utilized. In ArcViz, a non-linear mapping into a two-dimensional plane is performed on the high-dimensional data (clinical information) by considering variables as arcs. Each multi-dimensional data point that includes clinical information of each patient is mapped as a point inside a circular convex hull. The arcs of this circle represent each variable, and new dimensional anchors (points) are calculated between these arcs to determine the location of the mapped point as well as the covering area of each arc. All points are normalized on the axes between the center [(x, y) = (0, 0)] and each calculated anchor point that is located on the arcs. The projection of the clinical variables was then optimized using a genetic algorithm based on linear discriminant analysis (LDA) fitting and the nearest centroid (NC) accuracy of the fitting was calculated accordingly.

Three properties are associated with the mapping process in ArcViz: (1) the larger the value of a variable inside the multi-dimensional data point, the closer the mapped point will be toward the anchor point located on the arc representing this variable; (2) the mapped point gets closer to the center if its data point values across the variables are similar; and (3) the mapped point is determined from a combination of anchor points calculated on the arcs and mapped within their convex hull.

### Deep Learning Models

**Figure 1:**
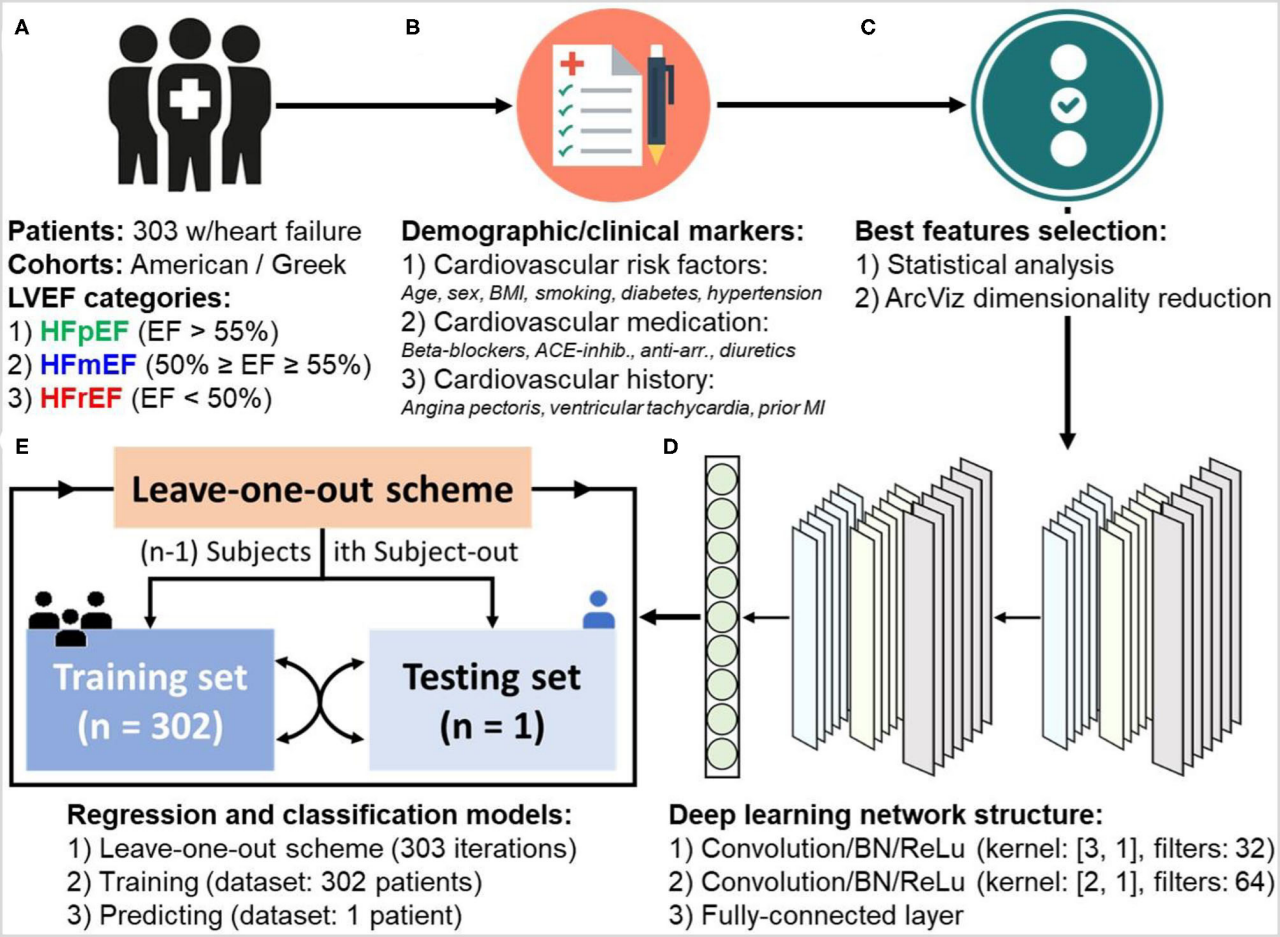
Workflow of developing deep learning-based regression and classification models for the prediction of left ventricular ejection fraction (LVEF) levels and categories in heart failure patients. The procedure goes through: **(A)** dataset collection (*n* = 303 patients), **(B)** patient information and demographic/clinical markers categorization, **(C)** statistical analysis of markers and dimensionality reduction using ArcViz (feature selection), **(D)** designing deep learning network structure, and **(E)** development of regression and classification models with leave-one-out training and predicting scheme.

To provide a complete prediction approach (Figure 1), two deep learning-based models for regression (level estimations) and classification (category labels) of LVEF were developed. The input of these models was 303 patient clinical information (Figure 1A) including the previously mentioned demographic and clinical markers (Figure 1B). The results of the statistical analysis as well as dimensionality reduction based on ArcViz were used as feature selection approaches to assist in determining the most important markers for a maximized performance within the proposed deep learning models (Figure 1C). Both models for regression and classification were structured as a deep learning network (Figure 1D) with convolutional neural networks (CNN). Two convolutional layers were utilized, each followed by batch normalization (BN) and rectified linear unite (ReLu), to extract characteristics contaminated within patients' clinical markers of every LVEF category. The two consecutive convolutional layers were with kernel sizes of (1, 3) and (1, 2), respectively, and with 32 filters and 64 filters, respectively. The development of the models included training and prediction phases (Figure 1E). In the training phase, both models were trained for 300 epochs with a mini-batch size of 64. The adaptive moment estimation (ADAM) was selected as the optimizer with a learning rate of 0.001, L2-regularization of 0.0001, and decay rate of 0.90. For the prediction phase, a leave-one-out scheme, where each subject is held out as the testing subject on each training iteration, was adopted. This scheme provides a prediction for every subject in the dataset, while at the same time maximizing the amount of data included within the trained models. It allows for treating each patient as a completely hidden testing set to the trained models, thus, slightly addressing any issues on the generality in the training and testing phases due to the lack of any external patient testing sets.

The performance of the regression model was evaluated based on the overall accuracy level, which was calculated as the agreement between the estimated and original LVEF with an accepted error of ±5%. Furthermore, the average root mean square error (RMSE) and correlation coefficient, alongside the Bland-Altman (44) (with mean ± 2 std) and correlation plots of the estimation process were determined. To evaluate the performance of the classification model, analysis of the confusion matrix of predictions as well as the receiver operating characteristic (ROC) curves and the corresponding area under the ROC (AUROC) was applied. Additional performance evaluation metrics including accuracy, sensitivity, specificity, precision, and F1-score.

## Results

### Clinical Characteristics of Patients

**Figure 2:**
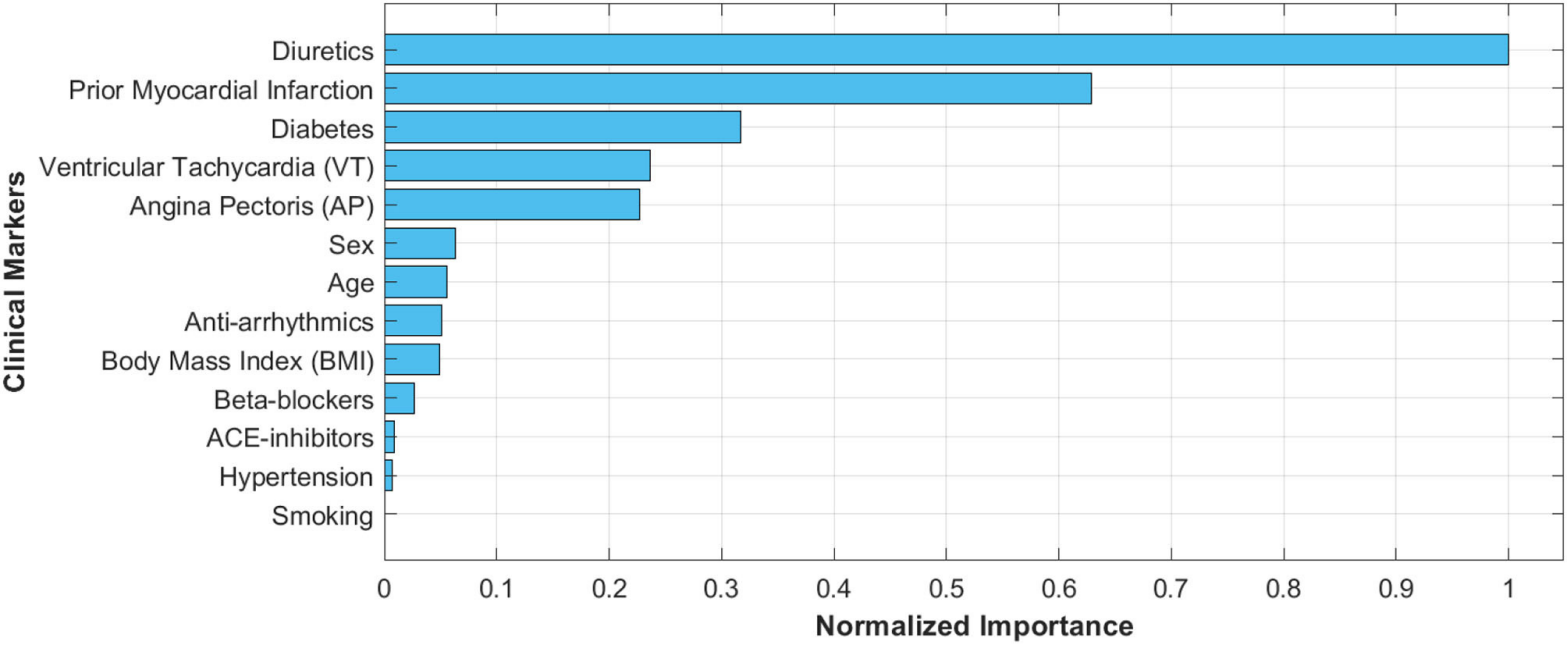
Normalized importance scores for the clinical markers used in the study in differentiating between the three LVEF categories. Importance scores were calculated using the Chi-squared (χ^2^) statistical test.

Patients included in this study had a median age of 58 years with an interquartile range of 50–65 years. Two hundred and fifty-eight patients were male (85.15%). Diabetes, diuretics medication, and prior MI showed significant differences in discriminating between the three LVEF categories (*p* < 0.001). Furthermore, for patients with diabetes, a significant difference was observed between HFpEF and HFrEF, whereas for diuretics medication and prior MI the significant differences were observed for HFpEF vs. HFrEF and HFpEF vs. HFmEF (*p* < 0.001). The complete clinical characteristics of the patient cohort is shown in Table 1 alongside the *p*-value calculations using linear regression fitting. The three aforementioned markers had the highest normalized importance scores using the Chi-squared (χ^2^) test as illustrated in Figure 2 (diuretics: 1.0, Prior MI: 0.63, and diabetes: 0.32). Additionally, VT and AP had relatively high scores with 0.24 and 0.23, respectively, with the remaining clinical markers being below 0.1.

**Table 1:** Clinical characteristics of the heart failure patients based on their left ventricular ejection fraction categories.

**Clinical variables**	**Overall subjects (*n* = 303)**	**LVEF categories**			***p*** **-value**			
		**HFpEF** **(*n* = 129)**	**HFmEF** **(*n* = 92)**	**HFrEF** **(*n* = 82)**	**HFpEF** **HFrEF**	**HFpEF HFmEF**	**HFmEF** **HFrEF**	**HFpEF** **HFmEF** **HFrEF**
LVEF, %	55 (46.5–63)	63 (60–70)	52.5 (50–55)	45 (40–47)	**<0.001**	**<0.001**	**<0.001**	**<0.001**
**Cardiovascular risk factors**								
Age, years	58 (50–65)	57 (38–64.5)	58.5 (52–68)	60.5 (50–66)	0.155	**0.034**	0.533	0.110
Male	258 (85.15)	108 (83.72)	76 (82.61)	74 (90.24)	0.181	0.828	0.147	0.237
BMI, kg/m^2^	27.28 (24.91–29.41)	27.12 (24.39–28.95)	27.22 (25.35–29.92)	27.68 (25.31–29.74)	0.159	0.166	0.945	0.133
Smoking	203 (67.00)	87 (67.44)	62 (67.39)	54 (65.85)	0.752	0.928	0.831	0.757
Diabetes	43 (14.19)	10 (7.75)	13 (14.13)	20 (24.39)	**<0.001**	0.127	0.086	**<0.001**
Hypertension	154 (50.83)	64 (49.61)	46 (50.00)	44 (53.66)	0.569	0.955	0.632	0.587
**Cardiovascular medication**								
Beta-Blockers	245 (80.86)	101 (78.30)	77 (83.70)	67 (81.71)	0.551	0.320	0.731	0.478
ACE-Inhibitors	113 (37.29)	47 (36.43)	33 (35.87)	33 (40.24)	0.555	0.932	0.580	0.611
Anti-Arrhythmics	12 (3.96)	3 (2.33)	4 (4.35)	5 (6.10)	0.164	0.400	0.605	0.166
Diuretics	114 (37.62)	24 (18.61)	51 (55.44)	39 (47.56)	**<0.001**	**<0.001**	0.302	**<0.001**
**Cardiovascular history**								
AP	186 (61.39)	89 (68.99)	46 (50.00)	51 (62.20)	0.311	**0.004**	0.107	0.189
VT	21 (6.93)	8 (6.20)	2 (2.17)	11 (13.42)	0.078	0.158	**0.005**	0.088
Prior MI	223 (73.60)	77 (59.69)	76 (82.61)	70 (85.37)	**<0.001**	**<0.001**	0.624	**<0.001**

*All values are represented as median (interquartile range) or n (%). Bold p-values show statistically significant differences (p < 0.050) amongst the selected categories. LVEF, Left ventricular ejection fraction; HFpEF, Heart failure with preserved ejection fraction; HFmEF, Heart failure with mid-range ejection fraction; HFrEF, Heart failure with reduced ejection fraction; BMI, Body mass index; ACE, Angiotensin-converting enzyme; AP, Angina pectoris; VT, Ventricular tachycardia; MI, Myocardial infarction*.

### ArcViz Representations of Clinical Markers

**Figure 3:**
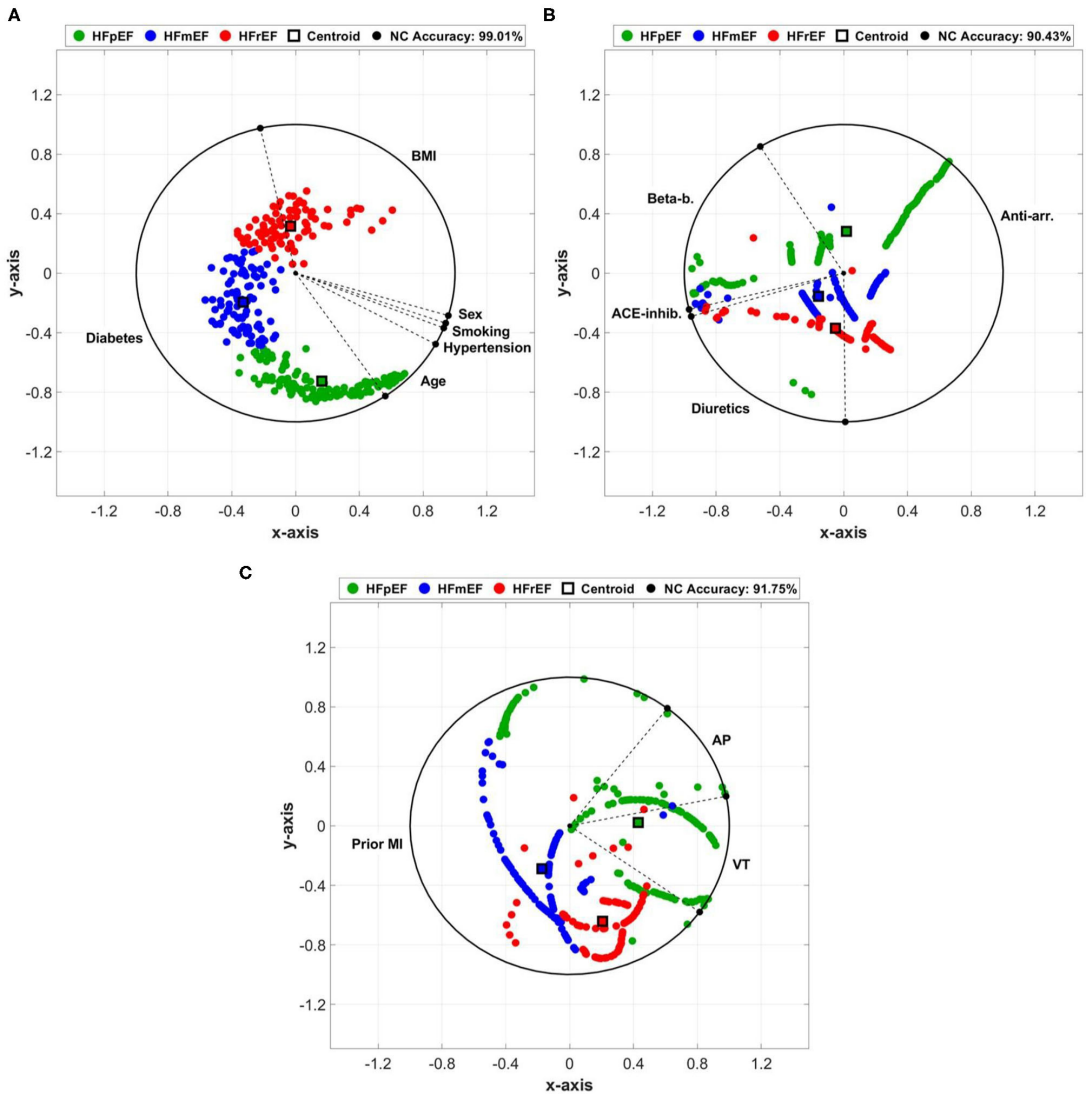
Arc visualization (ArcViz) and dimensionality reduction for: **(A)** cardiovascular risk factors, **(B)** cardiovascular medication, and **(C)** cardiovascular history clinical markers. The nearest centroid (NC) accuracy was calculated after optimizing linear discriminant analysis (LDA) fitting.

The projection of clinical markers on the ArcViz convex hull (Figure 3) yielded an average NC accuracy of 93.73%, distributed as 99.01, 90.43, and 91.75% for cardiovascular risk factors, cardiovascular medication, and cardiovascular history categories, respectively. For cardiovascular risk factors (Figure 3A), the three LVEF categories were perfectly separated with a large arc area for diabetes. This indicates the strong impact of diabetes on discriminating the three categories. Furthermore, although the centroids of HFpEF and HFmEF were located within diabetes, the centroid for HFrEF was located in the BMI region, which matches with the *p*-value observations of HFpEF vs. HFrEF in diabetes (Table 1—*p* < 0.001). It is worth noting that age had a greater effect on some HFpEF and therefore it was found to be significantly different for HFpEF vs. HFmEF as shown in Table 1 (*p* = 0.034). For cardiovascular medication (Figure 3B), a fair separation was obtained between the three LVEF categories associated with anti-arrhythmics and diuretics medication use. Both centroids of HFmEF and HFrEF were located within the diuretics arc region, with *p* < 0.001 (Table 1) observed between HFmEF and HFrEF compared to HFpEF, which was located mostly within the anti-arrhythmics arc region. Lastly, for cardiovascular history (Figure 3C), the stronger impact was due to the prior MI marker that had the lowest (*p* < 0.001; Table 1) for differences between HFpEF and HFmEF as well as HFpEF and HFrEF. The centroid of the HFpEF was located within the VT arc region, whereas both centroids of HFmEF and HFrEF were located within the prior MI arc region. The slight shift of the HFrEF category toward the VT biomarker arc area is reflected by the low (*p* = 0.005; Table 1) when compared to patients in the HFmEF group.

### Deep Learning Prediction of LVEF

**Figure 4:**
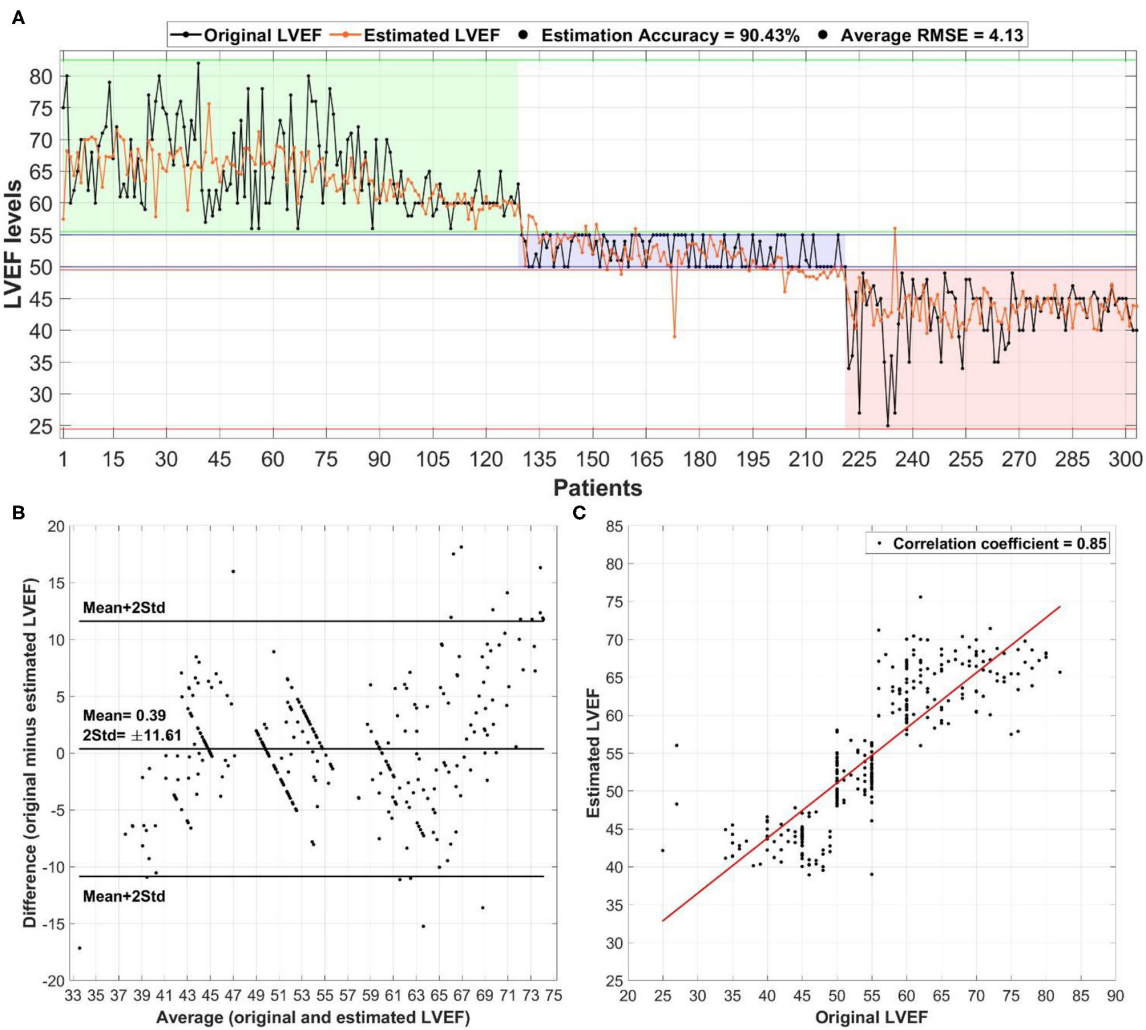
The overall performance of the deep learning-based regression model in estimating left ventricular ejection fraction (LVEF) levels in heart failure patients: **(A)** estimation of LVEF relative to the original levels alongside the overall accuracy and average root mean square error (RMSE), **(B)** Bland-Altman plot for the average vs. difference between the estimated and original LVEF levels with the mean ± 2 std difference level, and **(C)** correlation plot between estimated and original LVEF levels with the corresponding correlation coefficient.

**Figure 5:**
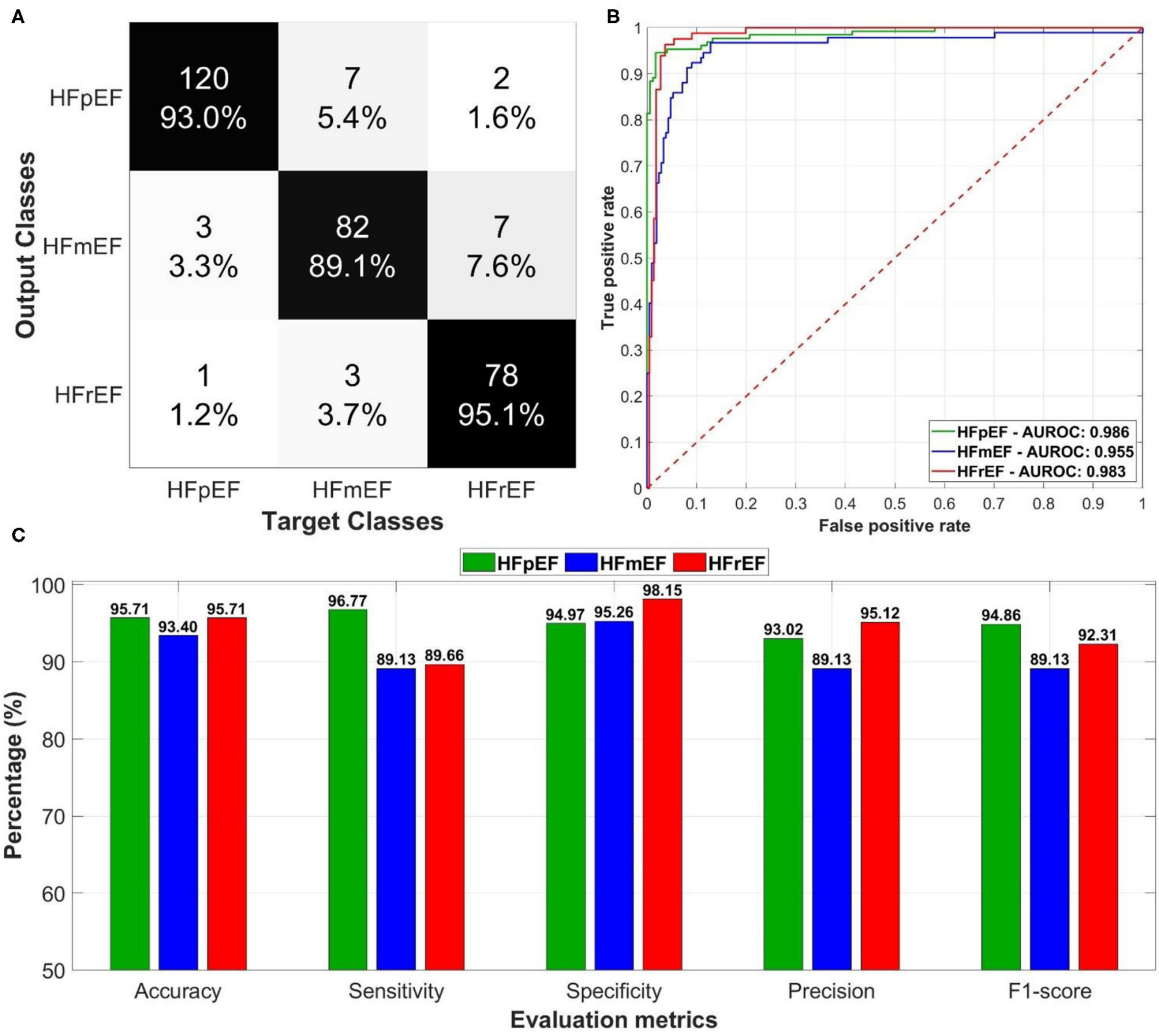
The overall performance of the deep learning-based classification model in predicting left ventricular ejection fraction (LVEF) categories in heart failure patients: **(A)** confusion matrix of the predictions for the output and target classes, **(B)** receiver operating characteristics (ROC) curves for each LVEF category with the corresponding area under the ROC curves, and **(C)** evaluation metrics including accuracy, sensitivity, specificity, precision, and F1-score.

Both deep learning models (regression and classification) were trained on the NVIDIA GeForce GTX 1070 graphics processing unit (GPU) of 8 GB display memory (VRAM). Training of each model required <1 min, while the prediction per-patient took <3 s. Both models were trained using the most important clinical markers (age, BMI, diabetes, anti-arrhythmics, diuretics, VT, and Prior MI) based on statistical significance and location of ArcViz centroids. The developed regression model (Figure 4A) successfully estimated patient LVEF levels with an overall accuracy of 90.43% (error: ±5%). Furthermore, the estimated LVEF levels had an average RMSE of 4.13 relative to the original LVEF levels. The Bland-Altman plot (Figure 4B) had a mean difference of 0.39 ± 11.61 between the estimated and original LVEF levels. Additionally, the correlation plot was skewed positively with an overall coefficient of 0.85 (Figure 4C). The classification model developed for this project (Figure 5A) efficiently predicted each LVEF category with a precision level of 93.00, 89.10, and 95.10% for HFpEF, HFmEF, and HFrEF, respectively.

The model resulted in an average AUROC of 0.975 (Figure 5B) distributed as 0.986 for HFpEF, 0.955 for HFmEF, and 0.983 for HFrEF. Furthermore, the model achieved high levels of performance (Figure 5C) in accuracy, sensitivity, specificity, precision, and F1-score (more than 89%).

To elaborate on the generality and performance of the proposed deep learning models, support vector machines (SVM) based on a radial basis function (RBF) kernel and generalized linear model (GLM) were used to estimate LVEF (regression) and predict HF categories (classification). The performance of both models are compared with the aforementioned deep learning results in Table 2. LVEF estimation accuracies using SVM and GLM models have reached 87.46% and 84.82%, respectively, which was outperformed by deep learning (90.43%). In addition, the RMSE had its lowest levels for deep learning (4.13) compared with SVM (4.38) and GLM (5.11). In predicting HF categories, the overall accuracy reached 88.45 and 84.14% for SVM and GLM, respectively, whereas it reached 90.10% in deep learning. It is worth noting that both models had high precision levels in HFrEF prediction with a 94.12% using SVM and 96.34% using GLM. However, they both had lower performance metrics (sensitivity, 1-specificity, and precision) than deep learning in discriminating between HFpEF and HFmEF.

**Table 2:** Performance comparison between deep learning support vector machines, and generalized linear model in estimating LVEF levels and predicting HF categories.

**Trained models**	**Regression (LVEF estimations)**				**Classification (Categories prediction)**									
	**Accuracy %**	**RMSE**	**Bland-Altman Mean ± 2 Std**	**Correlation Coefficient**	**HFpEF**			**HFmEF**			**HFrEF**			**Overall**
					**Sens. %**	**1-Spec. %**	**Prec. %**	**Sens. %**	**1-Spec. %**	**Prec. %**	**Sens. %**	**1-Spec. %**	**Prec. %**	**Acc. %**
Deep learning	90.43	4.13	0.39 ± 11.61	0.85	96.77	5.03	93.02	89.13	4.74	89.13	89.66	1.85	95.12	90.10
Support vector machine	87.46	4.38	0.14 ± 11.89	0.82	94.78	10.64	84.50	81.00	5.42	88.04	88.64	1.76	94.12	88.45
Generalized linear Model	84.82	5.11	0.05 ± 14.04	0.75	88.14	13.51	80.62	76.09	10.43	76.09	84.95	1.43	96.34	84.14

*LVEF, Left ventricular ejection fraction; HFpEF, Heart failure with preserved ejection fraction; HFmEF, Heart failure with mid-range ejection fraction; HFrEF, Heart failure with reduced ejection fraction; RMSE, Root mean square error; Sens, Sensitivity; Spec, Specificity; Prec, Precision; Acc, Accuracy*.

## Discussion

In this study, we demonstrated the significance of utilizing deep learning as a tool to estimate LVEF levels in HF patients as well as to categorize HF patients in accordance with their LVEF levels, offering an easily used and automated assistive tool for everyday clinical practice. The adopted narrower band for the HFmEF highlights that even slightly reduced values of LVEF can have an effect on heart rhythm and hence change in patient condition. Therefore, it was essential to employ versatile criteria for various cohorts in order to enable the adaptive analysis of the collected patient data. In addition, the ability to use automated deep learning-based trained models could save crucial time in clinical circumstances. In addition, these models may be able of aiding in the clinical decision making in HF assessment by going through available patient information with less dependence on medical experts. Only few studies have identified and discussed clinical information that may be capable of classifying HFpEF, HFmEF, and HFrEF patients statistically as well as from a machine learning-based perspective. To fill this gap, in the current study important clinical markers were first statistically identified and then projected into a novel arc radial visualization (ArcViz). Furthermore, a complete deep learning approach was developed that ensures higher levels of performance for automatic estimation of LVEF levels and differentiation between the three HF categories from clinical profiles only.

### Clinical Markers Significance

Thirteen clinical markers often found in patient profiles were evaluated statistically as well as through a new dimensionality reduction approach (ArcViz). Among these markers, 7 were found to be important in classifying HF patients based on LVEF.

For cardiovascular risk factors, age was found to be an important marker in differentiating between HFpEF and HFmEF. However, HFpEF patients were more skewed toward the age region in ArcViz analysis in agreement with previous studies that have found that HFmEF patients were younger in age and closer to HFrEF in comparison to the HFpEF patients (24, 45, 46). Furthermore, BMI, although not significantly different, was better in differentiating HFrEF from the other two groups when applying ArcViz. Of interest and in agreement with our study, several previous studies (47, 48) suggested that higher BMI often associated with HFrEF patients (was beneficial to this patient group), as higher BMI may counteract catabolism inflammation and stress hormone activation in the HFrEF group. However, a high body weight in HFpEF patients is usually strongly associated with HF, causing this patient group to be at higher risk of developing further adverse cardiac events. Additionally, it was shown that BMI does not play a critical role in HF progression apart from the HFrEF category which has a higher 30-day mortality (49). In this study, the minimum-maximum range for BMI of HFpEF patients was 19.7–36.3 kg/m^2^ with 21 obese patients (>30 kg/m^2^). For HFmEF patients, the range was 18.0–37.7 kg/m^2^ with 23 obese patients. For HFrEF patients, the range was 20.8–37.9 30 kg/m^2^ with 17 obese patients. These ranges fit with the usual BMI range (20–40 kg/m^2^) reflecting normal spread of BMI values across the included patients in the three LVEF categories. This could elaborate on the insignificance found using statistical analysis considering the narrow LVEF ranges in the three categories. Lastly, diabetes was found to be significantly different as well as the best in characterizing the three LVEF categories in ArcViz. Our findings show that the three LVEF categories can be better discriminated according to patients' diabetes diagnosis. All-cause mortality rates are reported to increase in diabetic HFrEF relative to HFpEF (50–52). However, patients within HFpEF and HFmEF groups showed higher burden of diabetes than HFrEF patients (53).

Important markers associated with cardiovascular medication included anti-arrhythmics and diuretics medication. Patients with HFpEF had a higher intake of both medications followed by HFrEF and lastly HFmEF. This shows a distinct medication procedure between the three LVEF categories using these two medications. The literature reports that HFpEF patients are more prone to atrial fibrillation, and thus, anti-arrhythmics medication is usually needed. Further, they were more likely to undergo repeated ablations compared to the HFrEF group (54, 55). This elaborates on the high number of patients taking anti-arrhythmics medication observed in this study for the HFpEF category with a better representation between HFpEF and HFrEF in the ArcViz analysis. In addition, use of diuretics medication was found to be highly discriminant between HFpEF and the other two LVEF categories. Previous studies reported that the prevalence of diuretics intake among HFmEF patients was found to be less than the prevalence in the HFpEF and HFrEF in agreement with the current study (45). Furthermore, diuretics are widely used in HFpEF and HFrEF patients to prevent symptoms of congestion in HF (56). This information supports the findings of this study by considering diuretics as a highly favored clinical marker in classifying patients into one of the three LVEF categories.

Lastly, in cardiovascular history, the best marker was the occurrence of prior MI. In a few previous studies (14, 57), a greater number of prior MI was observed in HFrEF compared to HFpEF. However, this could be due to the drop of LVEF levels in the HFrEF patients included in these studies, as a higher rate of prior MI is usually recorded if LVEF levels are <40% (58), which was found in very few cases in our study. In addition, VT was found to be the second most important cardiovascular history marker in characterizing the three LVEF categories using ArcViz analysis, especially the HFpEF, as well as being significant in discriminating HFrEF from the two other categories. This relates to the higher burden of VT observed in patients with HFpEF over patients in the other LVEF categories (59, 60).

It is worth mentioning that sex (male/female) was not found to be significant nor effective in characterizing any LVEF category in ArcViz analysis. However, this could be due to the high number of male patients enrolled in this study and needs to be further investigated as sex has been shown to be a factor in the prevalence of HF (60–63).

### Deep Learning as an Assistive Tool

Our study suggests deep learning as an assistive tool that could be capable of automatically reading and extracting characteristics from the clinical records of HF patients. In comparison with machine learning, our trained models allow for training on deeply extracted attributes between patients of each LVEF category. Thus, it was less biased than feature engineering techniques often used in conventional machine learning algorithms. Our novel deep learning models may assist clinicians based on the automated estimation of LVEF as well as the accurate classification into one of the three main HF categories (64). Furthermore, the models estimate and predict LVEF based on the cardiovascular risk factors, medication, and history. Additionally, the high levels of performance achieved in our deep learning models suggest the potential of relatively simple, yet effective, artificial intelligence algorithms in identifying certain clinical characteristics that differentiate between LVEF categories that may not be possible in conventional approaches. Although deep learning has outperformed other machine learning models in this work including SVM and GLM, further testing on external patient cohorts are still needed to elaborate further on the general validity of the achieved performance.

### Limitations

Although our study shows that deep learning-based models have performed efficiently in LVEF predictions, it has a number of shortcomings. First, we have utilized 13 features (the 7 most important ones were selected later) that were available in the databases used in this study. However, additional markers need to be further investigated, especially echocardiographic attributes, i.e., left ventricular diastolic and systolic dimensions (LVDD and LVDS), to provide more information on their effects on LVEF predictions. Moreover, even though the dataset used in this study combined patients from American and Greek populations, the trained models should be tested further on wider sets of patients to ensure additional generality of the performance. Future studies should focus on using external validation sets from different patient cohorts to imply general validity of the trained models. In addition, the proposed models were trained and tested on a specific range for each LVEF category as recommended by the ASE/EACVI guidelines. Despite having a narrower border-line for the HFmEF (50% ≤ EF ≤ 55%), further studies of other LVEF guidelines and recommended LVEF category ranges may enhance the effectiveness of deep learning in LVEF predictions. Furthermore, validation on longitudinal data needs to be undertaken to identify efficacy of intervention over time based on the current models. Lastly, patients cohort in this study includes a much higher percentage of males compared to females. In addition, the median BMI of the included patients was 27.28 kg/m^2^ indicating overweight but not obese subjects with a narrow BMI range between the three LVEF categories. Future studies with cohorts differing with reference to all demographic categories including BMI are needed in order to demonstrate the efficacy of the proposed methods in all possible populations/clinical scenarios with narrow or wide ranges for clinical information across LVEF categories.

### Conclusions

Overall, our novel deep learning-based models showed high levels of performance in automatically estimating LVEF levels as well as classifying HF patients into one of the three LVEF categories, suggesting it as a promising assistive tool in clinical settings. The developed approach may lead to a better understanding, from a machine learning (or deep learning) perspective, of the clinical variables most suitable for discriminating HFpEF, HFmEF, and HFrEF. The proposed study is to extend the applicability of use of LVEF to communities where the required instruments are not available due to economic hardship or lack of clinical expertise. Future research can add additional demographic and clinical information to the deep learning models alongside clinical profiles for an even better performance and understanding of the differences between each LVEF category. Our outcomes may also facilitate the development of a model for the prediction of the HF phenotype or its changes during the followed therapy of HF, offering a versatile tool for the further exploration of disease pathophysiology or the objective assessment of the different therapeutic schemes in future patients with HF.

## Data Availability Statement

Patient data used in this study will be provided by contacting the corresponding author upon reasonable request. Deep learning networks, final trained models, and regression/classification main code (including data preparation) are available at: https://github.com/malkhodari/Alkhodari_frontiers. Requests to access these datasets should be directed to Mohanad.alkhodari@ku.ac.ae.

## Ethics Statement

Patients enrollment protocol in the Intercity Digital Electrocardiography (ECG) Alliance (IDEAL) study was conducted according to Title 45, U.S. Code of Federal Regulations, Part 46, protection of human subjects (revised: November 13, 2001 – effective: December 13, 2001) and in accordance with the Declaration of Helsinki. Furthermore, the research subject review board of the University of Rochester approved the IDEAL protocol. All patients provided a consent form before participating in the study. Furthermore, the enrollment protocol of patients for the PRESERVE EF study was approved by the ethics committee at each of the seven selected cardiology departments at Greece and was endorsed by the Hellenic Society of Cardiology. All patients signed a consent form prior to enrollment in the study at each cardiology department.

## Author Contributions

MA, HJ, LH, and AHK: designed research idea. MA: performed literature search, algorithm implementation, statistical analysis, and deep learning modeling. HJ and AK: advised on the categorization of clinical information in patient profiles. SS and AHK: clinical data acquisition and preparation. MA: wrote the initial draft of the manuscript. HJ, AK, SS, LH, and AHK: edited the final version of the manuscript. All authors reviewed and agreed on the manuscript and ensured that any questions on the work are appropriately resolved.

## Funding

This work was supported by a grant (award number: 8474000132) from the Healthcare Engineering Innovation Center (HEIC) at Khalifa University, Abu Dhabi, UAE, and by grant (award number: 29934) from the Department of Education and Knowledge (ADEK), Abu Dhabi, UAE.

## Conflict of Interest

The authors declare that the research was conducted in the absence of any commercial or financial relationships that could be construed as a potential conflict of interest.

## Publisher's Note

All claims expressed in this article are solely those of the authors and do not necessarily represent those of their affiliated organizations, or those of the publisher, the editors and the reviewers. Any product that may be evaluated in this article, or claim that may be made by its manufacturer, is not guaranteed or endorsed by the publisher.
